# Biochemical Characterization and Complete Conversion of Coenzyme Specificity of Isocitrate Dehydrogenase from *Bifidobacterium longum*

**DOI:** 10.3390/ijms17030296

**Published:** 2016-02-26

**Authors:** Shi-Ping Huang, Hong-Mei Cheng, Peng Wang, Guo-Ping Zhu

**Affiliations:** The Research Center of Life Omics and Health and Anhui Provincial Key Laboratory of the Conservation and Exploitation of Biological Resources, Anhui Normal University, Wuhu 241000, Anhui, China; huangsp2014@gmail.com (S.-P.H.); chengmei325111@163.com (H.-M.C.); wangpeng1219@yahoo.com (P.W.)

**Keywords:** *Bifidobacterium longum*, isocitrate dehydrogenase, biochemical characterization, coenzyme specificity determinants, kinetics

## Abstract

*Bifidobacterium longum* is a very important gram-positive non-pathogenic bacterium in the human gastrointestinal tract for keeping the digestive and immune system healthy. Isocitrate dehydrogenase (IDH) from *B. longum* (*Bl*IDH), a novel member in Type II subfamily, was overexpressed, purified and biochemically characterized in detail. The active form of *Bl*IDH was an 83-kDa homodimer. Kinetic analysis showed *Bl*IDH was a NADP^+^-dependent IDH (NADP-IDH), with a 567- and 193-fold preference for NADP^+^ over NAD^+^ in the presence of Mg^2+^ and Mn^2+^, respectively. The maximal activity for *Bl*IDH occurred at 60 °C (with Mn^2+^) and 65 °C (with Mg^2+^), and pH 7.5 (with Mn^2+^) and pH 8.0 (with Mg^2+^). Heat-inactivation profiles revealed that *Bl*IDH retained 50% of maximal activity after incubation at 45 °C for 20 min with either Mn^2+^ or Mg^2+^. Furthermore, the coenzyme specificity of *Bl*IDH can be completely reversed from NADP^+^ to NAD^+^ by a factor of 2387 by replacing six residues. This current work, the first report on the coenzyme specificity conversion of Type II NADP-IDHs, would provide better insight into the evolution of NADP^+^ use by the IDH family.

## 1. Introduction

Isocitrate dehydrogenase (IDH) belongs to an ancient and ubiquitous metal-dependent β-decarboxylating dehydrogenase family that plays critical roles in amino acid biosynthesis, vitamin production and energy metabolism [1,2,3]. IDHs are key enzymes in the tricarboxylic acid cycle (TCA cycle) that catalyze the oxidative decarboxylation of isocitrate to α-ketoglutarate (α-KG) and CO_2_ with NAD^+^ or NADP^+^ as a coenzyme [4]. According to the different coenzyme dependencies, IDHs play a variety of roles *in vivo*. NAD^+^-dependent IDH (NAD-IDH, EC 1.1.1.41) provides the first connection between TCA cycle and electron-transport pathway by producing NADH, which participates in energy metabolism [5]. NADP^+^-dependent IDH (NADP-IDH, EC 1.1.1.42) generates NADPH, which provides the reducing power for biosynthesis, maintains the redox state of the cell, and takes part in CO_2_ assimilation. Therefore, NADP-IDH is essential in glutathione metabolism, fatty acids and steroids biosynthesis, and cellular antioxidation systems [6,7,8]. Recently, increasing attention has been paid to human NADP-IDHs. It has been reported that the mutations at some active sites can confer a new function on NADP-IDHs to reduce α-ketoglutarate to 2-hydroxyglutarate, which correlates closely with the incidence of tumors [9,10,11], such as gliomas, the most common type of human brain cancers.

Based on the phylogenetic analysis, IDHs can be divided into three subfamilies: Type I IDHs, Type II IDHs and monomeric IDHs [12]. Most bacterial homodimeric NAD(P)-IDHs, homotetrameric NAD-IDHs and mitochondrial heteroligomeric NAD-IDHs are clustered into the Type I subfamily. The homodimeric NADP-IDHs from eukaryotes (in cytoplasm and mitochondria) and some bacterial NADP-IDHs are categorized into the Type II subfamily. It was noted that, recently, several algae IDHs were found to be Type II NAD-IDHs, whose coenzyme specificity can be completely converted from NAD^+^ to NADP^+^ by rational mutagenesis [12,13]. All monomeric enzymes, either NAD^+^ or NADP^+^-dependent, fall into the third subfamily. Type II NADP-IDHs from eukaryotes, such as yeast, pig and human have been extensively studied, including biochemical properties, the crystal structures and catalytic mechanisms [14,15,16]. On the contrary, the information of Type II NADP-IDHs from bacteria is very limited and only a few of them have been preliminarily characterized.

Furthermore, it was proposed that NAD^+^ use is an ancestral trait and NADP^+^ use by bacterial IDHs arose on or about the time that eukaryotic mitochondria first appeared, some 3.5 billion years ago [17]. The switch in coenzyme dependency from NAD^+^ to NADP^+^ by IDHs was an ancient adaptation for bacterial survival on energy-poor compound (such as acetate). This hypothesis has been proved by the competition experiment in the laboratory for NADP^+^-dependent IDH from *Escherichia coli* (*Ec*IDH), a typical member of Type I IDHs subfamily [17]. It is an interesting question that whether the selective mechanism of this ancient adaptation is also suitable to Type II IDHs subfamily , because the sequence similarity between two IDHs subfamilies is very low (<20%).

*Bifidobacterium longum*, a gram-positive and non-pathogenic bacterium, is one of the most popular probiotics in various dairy products to provide enormous health benefits for the healthy human gastrointestinal system, such as improving lactose tolerance, preventing diarrhea and inhibiting pathogen colonization [18,19,20,21]. Several studies have shown that *B. longum* plays a key role in modulating the immune system [22,23] and has certain guiding significance to cancer gene therapy [24,25].

In this work, the enzymology of a homodimeric IDH from *B. longum* (*Bl*IDH) in Type II subfamily was investigated in detail. In addition, the coenzyme specificity of *Bl*IDH was converted from NADP^+^ to NAD^+^ by site-directed mutagenesis, which may provide useful clues to explore the acquired cause of NADP^+^ dependency by Type II IDHs.

## 2. Results

### 2.1. Sequence Alignment

**Figure 1:**
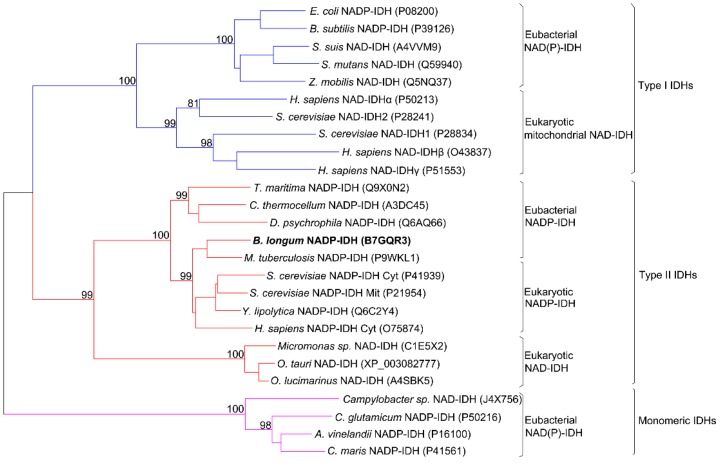
Phylogenetic tree of 26 isocitrate dehydrogenases (IDHs). A neighbor-joining tree with 500 bootstrap was created using MEGA 6.06. The GenBank accession numbers were noted in the parentheses.

The coding sequence of *Bl*IDH consisted of 1221 bp nucleotides with one open reading frame (ORF) encoding a protein of 406 amino acids. This enzyme showed a high level of sequence similarity to that of Type II NADP-IDHs, such as *Mycobacterium tuberculosis* IDH (73.5%), *Clostridium thermocellum* IDH (53.8%) and human cytosolic IDH (61.5%). Phylogenetic analysis was performed to further clarify the evolutionary relationship between *Bl*IDH and other IDHs. The result revealed that *Bl*IDH was clustered into the clade of Type II NADP-IDHs (Figure 1).

**Figure 2:**
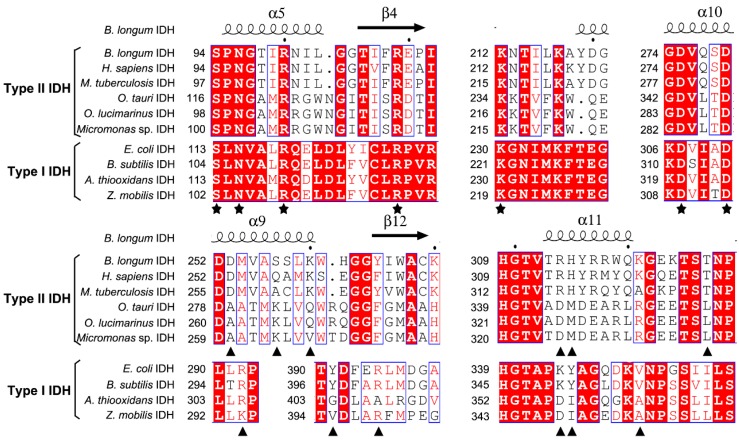
Structure-based protein sequences alignment of isocitrate dehydrogenase (IDH) from *B. longum* (*Bl*IDH) with other IDHs. The primary residues involved in substrate binding are indicated by pentagrams (★). The residues interact with the 2′-phosphate of NADP^+^ directly or indirectly are indicated by triangles (▲). The letters in blue boxes indicate conserved residues, and the white letters with red background in blue boxes indicate strictly conserved residues. The black letters in white boxes indicate similarity. The structure of *Bl*IDH was generated by SWISS-MODEL server. The figure created by ESPript 3.0.

In order to evaluate potential substrate and coenzyme-binding sites, multiple sequence alignments were performed (Figure 2). All amino acids involved in substrate binding are highly conserved in both Type I and II subfamilies, but the residues responsible for coenzyme binding are significantly different in the two subfamilies. When compared with NADP-IDH in the Type II subfamily, Arg314 and His315 in the binary complex of human cytosolic NADP-IDH (*Hc*IDH) are considered to be the major determinants of coenzyme specificity, which form salt bridges with the 2′-phosphate group of NADP^+^ [15].

**Figure 3:**
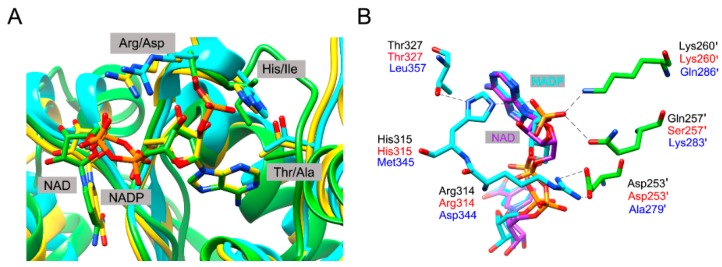
Comparison of the NADP^+^-binding sites among the human cytosolic NADP-IDH (*Hc*IDH), *Bl*IDH, *Acidithiobacillus thiooxidans* NAD-IDH (*At*IDH) and *Ostreococcus tauri* NAD-IDH (*Ot*IDH). (**A**) Overlay of the subunits of *Hc*IDH (yellow, PDB code: 1T0L), modelled *Bl*IDH (cyan) and *At*IDH (green, PDB code: 2D4V) highlighting the selected coenzyme binding sites in these three IDHs. The NADP^+^ molecule (with yellow C atoms) and NAD^+^ molecule (with green C atoms) were represented by the stick. The model of *Bl*IDH was generated by SWISS-MODEL server; (**B**) A close-up view showing the selected residues involving in NADP^+^ binding in *Hc*IDH (labelled by black). The equivalent residues in *Bl*IDH, targeted by site-directed mutagenesis, and in *Ot*IDH were labelled by red and blue, respectively.

The corresponding residues are completely conserved in *Bl*IDH (Arg314 and His315). In the quaternary complex of *Hc*IDH (Figure 3), the side chains of Gln257′ and Lys260′ (the prime indicates the other subunit of the homodimer) form hydrogen bonds with the 2′-phosphate group of NADP^+^, which are homologous to Ser257′ and Lys260′ in *Bl*IDH [15]. Furthermore, Arg314 in *Hc*IDH forms a salt bridge with Asp253′ (equivalent to Asp253′ in *Bl*IDH) and interacts with Arg249′ and Gln257′ [15]. As compared with NAD-IDH, Arg314 and His315 in *Bl*IDH are replaced by Asp357 and Ile358 in *Acidithiobacillus thiooxidans* NAD-IDH (*At*IDH) from Type I subfamily [26] and Asp344 and Met345 in *Ostreococcus tauri* NAD-IDH (*Ot*IDH) from Type II subfamily [13]. Asp253′, Ser257′ and Lys260′ in *Bl*IDH are substituted with Ala279′, Lys283′ and Gln286′ in *Ot*IDH, as shown in Figure 3.

### 2.2. Expression and Purification

**Figure 4:**
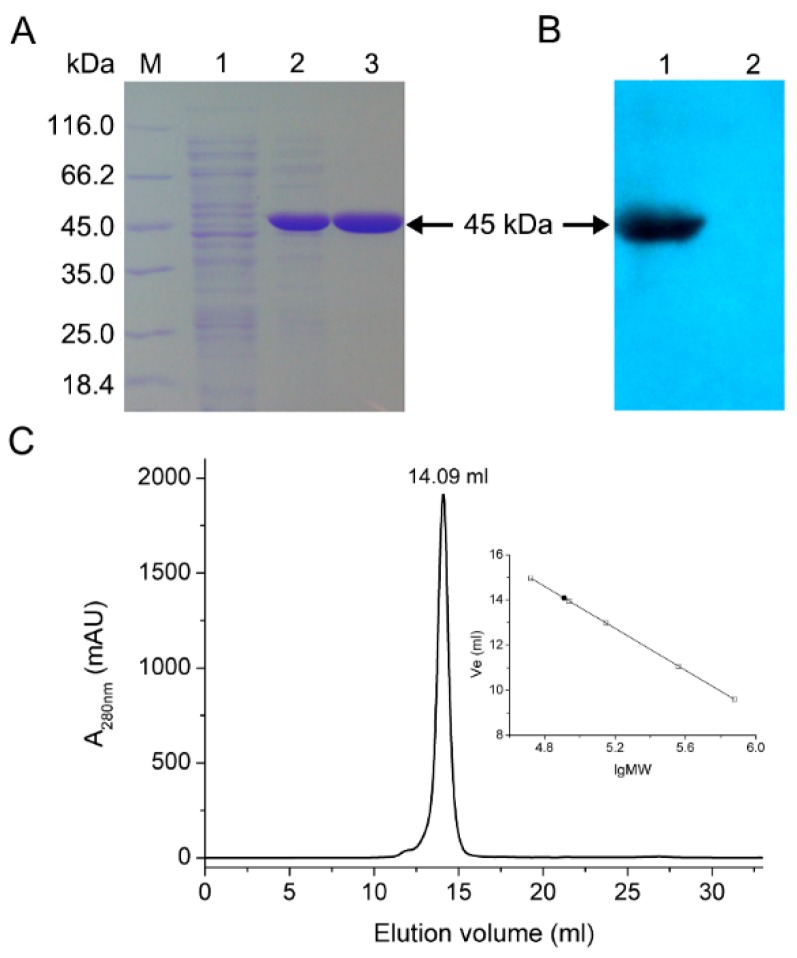
Overexpression and molecular mass determination of *Bl*IDH. (**A**) SDS-PAGE analysis. M, molecular mass marker; lane 1, crude extract from cells transformed by pET-28b(+) with IPTG treatment; lane 2, crude extract from cells transformed by recombinant plasmid pET-*Bl*IDH with IPTG treatment; lane 3, purified *Bl*IDH; (**B**) Western blot analysis. Lane 1, purified *Bl*IDH; lane2, negative control, crude extract from cells transformed by pET-28b(+) with IPTG treatment; (**C**) Size exclusion chromatography (SEC) analysis of *Bl*IDH.

Recombinant *Bl*IDH with 6×His tag was successfully heterologously expressed in *E. coli* Rosetta (DE3) and purified to homogeneity by Co^2+^ affinity chromatography. Molecular mass of the recombinant protein was determined to be approximately 45 kDa by SDS-PAGE, which compared well with the predicted value (45 kDa) (Figure 4A) and was confirmed by Western blotting by probing with anti-6×His antibody (Figure 4B). The oligomeric status of *Bl*IDH was determined by size exclusion chromatography (SEC), and a single symmetrical peak was observed (Figure 4C) while the native molecular mass of *Bl*IDH was calculated to be 83 kDa, suggesting that the native enzyme forms a homodimer in solution.

### 2.3. Kinetic Characterization

**Figure 5:**
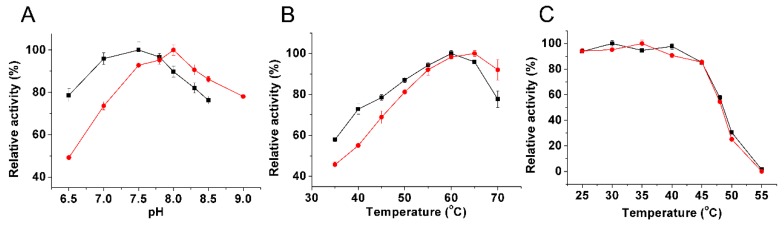
Effect of pH and temperature on the activity of *Bl*IDH in the presence of Mg^2+^ (●) and Mn^2+^ (■), respectively. (**A**) Effect of pH on the activity of *Bl*IDH; (**B**) Effect of temperature on the activity of *Bl*IDH; (**C**) Heat-inactivation profiles of the *Bl*IDH. The values indicate the means of at least three independent measurements.

The optimal pH values of the purified recombinant *Bl*IDH were 7.5 with Mn^2+^ and 8.0 with Mg^2+^ (Figure 5A), similar to the homodimeric NADP-IDH from *Leptospira interrogans* (pH 7.0 with Mn^2+^ and pH 8.0 with Mg^2+^) [27], but apparently lower than the monomeric IDHs from *Corynebacterium glutamicum* (pH 9.0 with Mg^2+^) [28] and *Chlorobium limicola* (pH 9.0 with Mg^2+^) [29].

The recombinant *Bl*IDH exhibited the maximal activity around 60 and 65 °C in the presence of Mn^2+^ and Mg^2+^, respectively (Figure 5B), similar to that of *L. interrogan* IDH (60 °C with Mn^2+^ and Mg^2+^) [27], but higher than those of NAD-IDHs from *Congregibacter litoralis* (35 °C with Mn^2+^ and Mg^2+^) [30] and *Zymomonas mobilis* IDH (55 °C with Mn^2+^ and Mg^2+^) [31]. Heat-inactivation studies revealed that the recombinant *Bl*IDH was stable under 45 °C, but rapidly lost activity above this temperature, and only 50% activity remained after a 20 min incubation at 48 °C (Figure 5C).

The effects of 11 metal ions on *Bl*IDH activity were also examined. The activity of recombinant *Bl*IDH was entirely dependent on the presence of a divalent cation, such as Mn^2+^, the most effective activator for *Bl*IDH catalysis (Table 1). However, several divalent metal ions, including Ni^2+^, Co^2+^, Zn^2+^, Cu^2+^ and Ca^2+^, inhibited the activity in the presence of Mn^2+^ or Mg^2+^, where Zn^2+^ showed the most inhibitory effects on *Bl*IDH activity.

The kinetic parameters for recombinant *Bl*IDH were determined in both NAD^+^- and NADP^+^-dependent forms (Table S1). The *K*_m_ values of *Bl*IDH for NADP^+^ were 19.45 μM with Mg^2+^ and 58.29 μM with Mn^2+^ (Figure S1A,B). The apparent *K*_m_ values of *Bl*IDH for NAD^+^ were over 184-fold and nine-fold higher than those for NADP^+^ in the presence of Mg^2+^ and Mn^2+^, respectively. As a result, the catalytic efficiency (*k*_cat_/*K*_m_) of *Bl*IDH showed 567-fold (Mg^2+^) and 193-fold (Mn^2+^) preference for NADP^+^ over NAD^+^, respectively. Evidently, *Bl*IDH has remarkably high coenzyme specificity toward NADP^+^ although it is a dual-specificity enzyme.

When compared with other homodimeric NADP-IDHs, the *K_m_* values for NADP^+^ of *Bl*IDH (19.45 μM with Mg^2+^) was similar to those of *L. interrogan* IDH (21.1 μM) [27] and *E. coli* IDH (17 μM) [32], but higher than that of *Rattus norvegicus* cytosolic IDH (11.5 μM) [33] and much lower than those of *Yarrowia lipolytica* IDH (59 μM) [34] and *Helicobacter pylori* IDH (176 μM) [35]. The *k*_cat_/*K*_m_ values for NADP^+^ of *Bl*IDH (1.87 s^−1^·μM^−1^ with Mg^2+^) was higher than that of *H. pylori* IDH (0.704 s^−1^·μM^−1^) [35], a member of Type I IDHs subfamily. When compared with other monomeric NADP-IDHs, in the presence of NADP^+^, the coenzyme affinity (1/*K*_m_) and the catalytic efficiency (1.87 s^−1^·μM^−1^ with Mg^2+^ and 0.83 s^−1^·μM^−1^ with Mn^2+^) of *Bl*IDH were quite a bit lower than those of monomeric IDHs, such as *C. glutamicum* IDH (21.75 s^−1^·μM^−1^) [28] and *Colwellia maris* IDH (8.9 s^−1^·μM^−1^) [36].

### 2.4. Switch of Coenzyme Specificity

Amino acid sequence alignment, homology modeling and structural analysis of Type II NADP-IDHs revealed that several amino acid residues in *Bl*IDH may interact with 2′-phosphate moiety of NADP^+^ directly or indirectly, including Arg314, His315, Thr327, Asp253, Ser257 and Lys260 (Figure 3). To switch coenzyme specificity of *Bl*IDH, these residues were selected as targets for site-directed mutagenesis. Here, only three mutants, single mutant R314D, triple mutant R314D/H315I/T327A and sextuple mutant D253A/S257K/K260Q/R314D/H315I/T327A, displayed detectable activity. Furthermore, circular dichroism (CD) spectra of mutants were very similar to that of wild type (Figure S2), indicating that these six mutations did not cause the conformational alterations of *Bl*IDH.

Kinetic analysis demonstrated that single mutant R314D resulted in an approximate 57-fold increase, from 19.45 to 1102 μM, in *K*_m_ for NADP^+^, with no significant change in *K*_m_ for NAD^+^ (Table 2). Furthermore, R314D showed a dramatic decrease (984-fold) in *k*_cat_/*K*_m_ toward NADP^+^ coupled with a 16.5-fold decrease in *k*_cat_/*K*_m_ toward NAD^+^. Thus, the single mutant R314D is still a dual-specificity enzyme with an approximate 9.5-fold preference for NADP^+^ over NAD^+^, although the wild-type *Bl*IDH exhibited 567-fold preference for NADP^+^. When another two amino acids (His315 and Thr327) were substituted with Ile315 and Ala327, the triple mutant showed no detectable activity for NADP^+^ but only for NAD^+^-linked reaction. However, the catalytic efficiency of R314D/H315I/T327A was very poor as compared to wild type (Table 2).

Mutations at six sites caused a 17-fold increase in *K*_m_ for NADP^+^ and a 28-fold decrease in *K*_m_ for NAD^+^ (Figure S1C,D). Furthermore, the catalytic efficiency of sextuple mutant was increased 1.2-fold for NAD^+^ while it retained only about 0.05% catalytic efficiency for NADP^+^. As a result, the sextuple mutant showed an approximately four-fold preference for NAD^+^ over NADP^+^ [(*k*_cat_/*K*_m_) ^NAD^/(*k*_cat_/*K*_m_) ^NADP^], which clearly indicated that the coenzyme specificity of *Bl*IDH was completely converted from NADP^+^ to NAD^+^ by a factor of 2387 via six-residue replacement.

## 3. Discussion

### 3.1. Coenzyme Specificity Determinants of BlIDH

IDH specificity is governed by residue interactions at three layers [37]. The “First Layer” residues directly contact with the unique 2′-hydroxyl and 2′-phosphate groups of NAD(P)^+^; The “Second Layer” residues are more distant amino acids, which can modulate the effects of the first group but not in contact with the unique cofactor moieties; The “Third Layer” residues are far from the cofactor binding site and form long-range interactions, which might promote the formation of a low-energy conformation.

Based on sequence alignment and available structural information, Arg314 and His315 in *Bl*IDH are considered to be the “First Layer” residues, two putative coenzyme specificity determinants, by directly interacting with 2′-phosphate group of NADP^+^. As expected, the single mutant R314D decreased the affinity for NADP^+^ by approximately 57-fold due to the removal of a salt bridge between Arg314 and 2′-phosphate group of NADP^+^ together with the removal of the electrostatic repulsion between negatively charged Asp and 2′-phosphate group of NADP^+^. When adjacent His315 was simultaneously mutated to Met or Ile, the resulting double mutant R314D/H315I and R314D/H315M lost activity completely. The loss in NADP^+^-dependent activity might result from the removal of favorable interactions between Arg314 or His315 and the 2′-phosphate group.

To achieve the NAD^+^-dependent activity, a third mutation (Thr327Ala) was introduced into the double mutant, creating R314D/H315I/T327A. Here, the residue Thr327 in *Bl*IDH is equivalent to Val351 in *Ec*IDH and therefore considered to be the “Second Layer” residue. The crystal structure of an *Ec*IDH mutant has revealed several essential indirect effects of Val351Ala on specificity alteration, such as to avoid obstructing adenine ring shift and to accommodate the favorable repacking in the adenosine-binding pocket [37]. The similar result has also been reported for *Haloferax volcanii* IDH that the replacement of Val by Ala was crucial for its engineered enzyme to obtain the activity with NAD^+^ as a coenzyme [38]. As a result, the triple mutant R314D/H315I/T327A restored partial activity for NAD^+^ (Table 3).

In order to improve the affinity and catalytic activity of R314D/H315I/T327A, a putative residue Asp253 forming hydrogen bond with Arg314 and two putative residues (Ser257 and Lys260) forming extra hydrogen bonds with 2′-phosphate group of NADP^+^ were engineered to the corresponding residues in NAD^+^-dependent *Ot*IDH (Ala279, Lys283 and Gln286), generating D253A/S257K/K260Q/R314D/H315I/T327A. Compared with the triple mutant (R314D/H315I/T327A), this sextuple mutant showed approximately a 34-fold increase in affinity and a 1.3-fold increase in catalytic activity, resulting in a 40-fold increase in catalytic efficiency for NAD^+^. With respect to the wild-type enzyme, the sextuple mutant displayed a 27.6-fold increase in affinity and a 1.2-fold increase in catalytic efficiency for NAD^+^. As a consequence, the sextuple mutant showed an approximately four-fold preference for NAD^+^ over NADP^+^ [(*k*_cat_/*K*_m_) ^NAD^/(*k*_cat_/*K*_m_) ^NADP^]. Therefore, the coenzyme specificity of *Bl*IDH was converted from a 567-fold preference for NADP^+^ to a four-fold preference for NAD^+^ through rational engineering of the major determinants for coenzyme specificity.

### 3.2. Evolutionary Implications for NADP^+^ Use by Prokaryotic Type II IDHs

Phylogenetic analysis and competition experiments have revealed that NADP^+^ use by bacterial IDHs of Type I subfamily evolved from NAD^+^ use about 3.5 billion years ago, and the cause of selection is the NADPH demand for bacterial adaptation to anabolic niches such as acetate [17]. For IDHs of Type I subfamily, their coenzyme specificity can be completely converted from NADP^+^ to NAD^+^ such as NADP-IDHs from *E. coli* and *H. volcanii* [32,38], or from NAD^+^ to NADP^+^ such as NAD-IDH from *Pyrococcus furiosus* [40].

The evolutionary origin of Type II subfamily is still obscure because all its members are NADP-dependent IDHs. As more and more genomic sequences have become available, some novel NAD-dependent IDHs are clustered into Type II subfamily after reconstructing the phylogenetic tree of IDHs. We deduce that the ancestor of Type II subfamily is also a NAD-dependent enzyme, the same as that of Type I subfamily. Several NAD-dependent IDHs have converted their coenzyme specificities from NAD^+^ to NADP^+^ by strategic amino acid replacements, such as NAD-IDHs from *Micromonas* sp. [12], *O. tauri* [13] and *C. litoralis* [30].

The information about successful inversion of coenzyme specificity in NADP-IDHs of Type II subfamily is very limited. In a previous report, only a single mutant of *Yarrowia lipolytica* NADP-IDH (*Yl*IDH, a Type II member), R322D, displayed poor activity for NAD^+^ [34]. In this work, the coenzyme specificity of *Bl*IDH mutant was converted from NADP^+^ to NAD^+^ by a factor of 2387, which is the first case for complete alteration of the coenzyme specificity of Type II NADP-IDHs. However, due to the absence of a putative gene encoding isocitrate lyase in *B. longum* genome, it is unclear whether the coenzyme specificity change of *Bl*IDH was also caused by the increased demand of NADPH under carbon starvation.

## 4. Experimental Section

### 4.1. Sequence Analysis

The X-ray structure of human cytosolic IDH (*Hc*IDH) was downloaded from the Protein Data Bank database (available online: http://www.rcsb.org/pdb/). SWISS-MODEL server (avaible online: http://swissmodel.expasy.org/) was employed to create the homology model of *Bl*IDH using *Hc*IDH structure (PDB code: 1T0L) as a template. Multiple protein sequence alignment was performed using ClustalW program and ESPript 3.0 web tool (available online: http://espript.ibcp.fr/ESPript/cgi-bin/ESPript.cgi). For phylogenetic analysis, 26 protein sequences (including *Bl*IDH) were aligned with ClustalW program. A Neighbor-Joining tree with 500 bootstrap was created via MEGA 6.06 program [41]. Protein structural figures were conducted using PyMOL [42] and UCSF Chimera [43].

### 4.2. Bacterial Srains and Reagents

The *E. coli* strains, DH5α and Rosetta (DE3), and plasmid pET-28b(+) were stored at low temperature in our laboratory. PrimeSTAR^TM^ HS DNA polymerase was purchased from TaKaRa Biotechnology Co., Ltd. (Dalian, China). Restriction endonucleases, T4 DNA ligase and protein molecular weight standards were obtained from Thermo Scientific (Shanghai, China).

### 4.3. Recombinant Plasmid Construction

Genomic DNA of *B. longum* subsp. *infantis* ATCC 15697 was bought from Leibniz Institute DSMZ-German Collection of Microorganisms and Cell Cultures (Braunschweig, Germany). According to the genomic sequence of *B. longum* (NCBI Reference Sequence: NC_011593.1), one pair of primers (Table S2) were designed to amplify the complete *icd* gene. Initial denaturing step of polymerase chain reaction (PCR) was 3 min at 95 °C, followed by 35 cycles of 95 °C for 30 s, 65 °C for 30 s, and 72 °C for 1 min and 30 s. After Nde I and Not I digestion, the PCR product was cloned into the expression vector pET-28b(+) which has a 6×His tag coding sequence upstream of the multiple cloning site. The *icd* gene in the recombinant plasmid pET-*Bl*IDH was sequenced (GenScript, Nanjing, China).

### 4.4. Site-Directed Mutagenesis

In order to identify the determinants and convert the coenzyme specificity of *Bl*IDH, six mutants were created by site-directed mutagenesis and overlap extension PCR technique [44]. The oligonucleotides for constructing the mutants were reported in Table S2. The mutated genes were constructed by sequential PCR steps. In the first step, two fragments containing the desired mutation were amplified with the following primers: *Bl*IDH-S and one of the antisense primers including the point mutation; one of the sense primers including the point mutation and *Bl*IDH-As. Then, the two overlapping fragments were purified and used as templates to amplify the full-length fragment using *Bl*IDH-S and *Bl*IDH-As. The final PCR products were digested by Nde I and Not I and ligated into the expression vector pET-28b(+). DNA sequencing was performed in both directions to verify all sequences of the mutated genes (GenScript, Nanjing, China).

### 4.5. Overexpression and Purification

The recombinant expression plasmids were transformed into *E. coli* Rosetta (DE3) strains, and grown overnight at 37 °C in Luria-Bertani (LB) medium supplemented with kanamycin and chloramphenicol to a final concentration of 30 μg/mL. The cultures were inoculated into 100 mL fresh LB media with the same antibiotics. When the optical density at 600 nm (OD_600_) of culture reached 0.4, the isopropyl-1-thio-β-d-galactopyranoside (IPTG) was added to a final concentration of 0.5 mM, followed by continuous growth at 20 °C for 20 h. The cells were collected by centrifugation at 5000× *g* for 5 min at 4 °C, and sonicated in lysis buffer (50 mM NaH_2_PO_4_, 300 mM NaCl, pH 7.8). The debris was removed by centrifugation at 12,000× *g* for 20 min at 4 °C. Finally, the recombinant proteins fused with 6×His-tag were purified with BD TALON metal affinity resin according to the manufacturer’s instructions (Clontech, Palo Alto, CA, USA).

### 4.6. SDS-PAGE and Western Blotting

The purity and molecular weight of *Bl*IDH were determined by 12% sodium dodecyl sulfate polyacrylamide gel electrophoresis (SDS-PAGE). For the Western blotting analysis, the recombinant enzymes were separated and transferred electrophoretically onto a nitrocellulose membrane. Then, the membrane was blocked for 1 h at room temperature in two solutions, TBS-T buffer (0.2% Tween-20, 150 mM NaCl and 50 mM Tris–HCl at pH 7.5) and 5% skim milk. Next, the membrane was immunoblotted with each antibody for 1 h, anti-6×His-tagpolyclonal antibody (cat#2365, Cell Signaling Technology, Danvers, MA, USA, 1:1000) followed by anti-rabbit IgG secondary antibody (cat#S3731, Promega, Madison, WI, USA, 1:2500). After that, the membrane was washed with TBS-T buffer for 10 min for three times. Finally, the chemiluminescence signal was observed by exposing the blot to X-ray film for an appropriate time period in a dark room.

### 4.7. Gel Filtration Chromatography

Molecular mass and oligomeric states of the recombinant *Bl*IDH were measured by gel filtration chromatography on a 10/300 Superdex 200 column (Amersham Biosciences, Freiburg, Germany) with equilibration buffer (50 mM NaH_2_PO_4_ and 150 mM NaCl at pH 7.2). The following standards were employed: Ovalbumin (44,000 Da), Conalbumin (75,000 Da), Aldolase (158,000 Da), Ferritin (440,000 Da) and Thyroglobulin (669,000 Da).

### 4.8. Circular Dichroism Spectroscopy

Circular dichroism (CD) spectra of the recombinant enzymes were measured with a Jasco model J-810 spectropolarimeter. Each sample were prepared in 75 mM Na_2_SO_4_ and 20 mM NaH_2_PO_4_ at pH 7.5 and then diluted to a final concentration of 0.2 mg/mL. The ellipticity (*θ*) was generated by averaging 3 scans of the protein solution between 195 and 260 nm at 0.5-nm increments. The mean molar ellipticity, [*θ*] (deg cm^2^ dmole^−1^), was calculated from [*θ*] = *θ*/10nCl, where the relationship has been described previously [45]. The *θ* is the measurement, n is the number of residues per subunit of protein (411 amino acids for *Bl*IDH), C is the molar concentration of the samples, and l is the cell path length (0.1 cm).

### 4.9. Enzyme Assays and Kinetic Characterization

The enzyme assay was described by Cvitkovitch *et al.* [46]. The activity assays were carried out at 25 °C in 1 mL reaction mixture containing 35 mM Tris-HCl at pH 8.0, 2 mM MgCl_2_ or MnCl_2_, 1 mM d,l-isocitric acid, and 0.5 mM NADP^+^ or 5 mM NAD^+^. The NAD(P)H production was monitored at 340 nm (ε340 = 6.22 mM^−1^·cm^−1^) using Cary300 UV-Vis spectrophotometer (Varian, Palo Alto, CA, USA). One unit of enzyme activity refers to 1 μM of NAD(P)H formed per minute. The enzyme concentration was detected by using Quick Start Bradford Protein Assay kit (Bio-Rad, Hercules, CA, USA).

To measure the *K*_m_ values of the recombinant enzymes for NAD(P)^+^, the concentration of isocitrate was fixed at 1.0 mM with varying coenzyme concentrations. Apparent *K*_m_ values for NAD(P)^+^ were calculated by nonlinear regression with GraphPad Prism 5.0 software (Prism, San Diego, CA, USA) and Origin 8.0 (OriginLab, Northampton, MA, USA). All kinetic parameters were measured in at least three independent experiments.

The effects of temperature, pH and metal ions on *Bl*IDH activity were measured using the standard assay method. The optimum pH was determined in the range of 6.0–9.0, and the optimal temperature was measured in the range of 35–60 °C. The half-life of *Bl*IDH was tested after incubation of enzyme aliquots at 25–55 °C for 20 min. The effects of metal ions on *Bl*IDH activity were also detected, including 2 mM monovalent metal cations (K^+^, Li^+^, Na^+^, and Rb^+^) and divalent metal cations (Mg^2+^, Mn^2+^, Co^2+^, Ca^2+^, Cu^2+^, Zn^2+^, and Ni^2+^).

## Figures and Tables

**Table 1:** Effects of metal ions on the activity of recombinant *Bl*IDH.

Metal Ions	Relative Activity (%)	Metal Ions	Relative Activity ^1^ (%)	Metal Ions	Relative Activity ^2^ (%)
Mn^2+^	100.00 ± 1.07	Mn^2+^	100.00 ± 1.07	Mg^2+^	100.00 ± 0.82
Mg^2+^	69.23 ± 0.82	Mn^2+^ + Mg^2+^	69.17 ± 0.41	Mg^2+^ + Mn^2+^	99.92 ± 0.41
Ni^2+^	5.67 ± 0.54	Mn^2+^ + Ni^2+^	47.42 ± 1.08	Mg^2+^ + Ni^2+^	43.37 ± 1.46
Co^2+^	14.30 ± 0.39	Mn^2+^ + Co^2+^	30.80 ± 0.40	Mg^2+^ + Co^2+^	39.58 ± 0.10
Zn^2+^	0	Mn^2+^ + Zn^2+^	5.19 ± 0.38	Mg^2+^ + Zn^2+^	11.02 ± 0.13
Cu^2+^	0	Mn^2+^ + Cu^2+^	31.04 ± 0.37	Mg^2+^ + Cu^2+^	61.38 ± 0.67
Ca^2+^	0	Mn^2+^ + Ca^2+^	62.33 ± 0.50	Mg^2+^ + Ca^2+^	14.06 ± 0.50
Li^+^	0	Mn^2+^ + Li^+^	103.83 ± 0.65	Mg^2+^ + Li^+^	100.52 ± 0.65
Rb^+^	5.26 ± 0.39	Mn^2+^ + Rb^+^	96.09 ± 0.57	Mg^2+^ + Rb^+^	105.77 ± 0.35
K^+^	4.89 ± 0.11	Mn^2+^ + K^+^	101.98 ± 0.30	Mg^2+^ + K^+^	103.75 ± 0.90
Na^+^	6.05 ± 0.43	Mn^2+^ + Na^+^	104.53 ± 0.15	Mg^2+^ + Na^+^	109.27 ± 0.18

^1^ The activity in the presence of Mn^2+^ alone is regarded as a 100% value for this column; ^2^ The activity in the presence of Mg^2+^ alone is regarded as a 100% value for this column. The values indicate the means of at least three independent measurements.

**Table 2:** The kinetic parameters of wild-type *Bl*IDH and mutants.

Residues at ^1^						NADP^+^			NAD^+^			Specificity (A/B)	Specificity (B/A)
253	257	260	314	315	327	*K*_m_ (μM)	*k*_cat_ (s^−1^)	*k*_cat_/*K*_m_ (s^−1^·mM^−1^) (A) ^2^	*K*_m_ (μM)	*k*_cat_ (s^−1^)	*k*_cat_/*K*_m_ (s^−1^·mM^−1^) (B) ^2^		
D	S	K	R	H	T	19.45 ± 2.6	36.38 ± 2.1	1870 ± 180	3584 ± 238.0	11.7 ± 5.2	3.3 ± 0.9	567	0.0017
(wild-type)													
*	*	*	D	*	*	1102 ± 92.6	2.15 ± 1.3	1.9 ± 0.7	3702 ± 117.1	0.88 ± 0.7	0.2 ± 0.1	9.5	0.105
*	*	*	D	I	*	-	-	-	-	-	-	-	-
*	*	*	D	M	*	-	-	-	-	-	-	-	-
*	*	*	D	M	L	-	-	-	-	-	-	-	-
*	*	*	D	I	A	-	-	-	4407 ± 196.7	0.41 ± 0.1	0.1 ± 0.01	-	-
A	K	Q	D	I	A	324.1 ± 27.9	0.307 ± 0.1	0.95 ± 0.05	130 ± 20.9	0.518 ± 0.03	4.0 ± 0.5	0.2375	4.21

“-” no apparently detectable activity. The values indicate the means of at least three independent measurements. ^1^ D, Aspartic acid; S, Serine; K, Lysine; R: Arginine; H, Histidine; T, Threonine; I, Isoleucine; M, Methionine; L, Leucine; A, Alanine; Q, Glutamine; *, denote the site without mutation; ^2^ A, *k*_cat_/*K*_m_^NADP^; B, *k*_cat_/*K*_m_^NAD^.

**Table 3:** Comparison of kinetic parameters between sextuple mutant and other IDHs.

Enzymes & Subfamilies	NAD^+^			NADP^+^			Specificity (A/B)	References
	*K*_m_ (μM)	*k*_cat_ (s^−1^)	*k*_cat_/*K*_m_ (s^−1^·μM^−1^) (A) ^c^	*K*_m_ (μM)	*k*_cat_ (s^−1^)	*k*_cat_/*K*_m_ (s^−1^·μM^−1^) (B) ^c^		
Type I NAD-IDH								
*S. suis* NAD-IDH (Mg^2+^) ^a^	233	41	0.176	9527	14	0.0015	117	[39]
*Z. mobilis* NAD-IDH (Mg^2+^)	312	88	0.282	8200	14	0.0017	165	[31]
Engineered *E. coli* NAD-IDH (Mg^2+^) ^b^	99	16.2	0.164	5800	4.7	0.00081	202	[37]
Type II IDH								
*C. litoralis* NAD-IDH (Mg^2+^)	262.6	36.7	0.140	-	-	-	-	[30]
*Micromonas* sp. NAD-IDH (Mg^2+^)	126	22.5	0.179	1827	1.4	0.0008	224	[12]
*O.* *lucimarinus* NAD-IDH (Mg^2+^)	136.6	60.6	0.444	2211	10.0	0.0045	99	[12]
*O. tauri* NAD-IDH (Mg^2+^)	226	59	0.261	3354	17	0.0051	51	[13]
*B. longum* NADP-IDH (Mg^2+^)	3584	11.70	0.003	19.45	36.4	1.87	0.0016	This study
Engineered *B. longum* NAD-IDH (Mg^2+^) ^b^	130	0.518	0.004	324.1	0.307	0.00095	4.21	This study
*Y. lipolytica* NADP-IDH (Mg^2+^)	-	-	-	59	72	1.22	-	[34]
Engineered *Y. lipolytica* NAD-IDH (Mg^2+^) ^b^	47,000	0.38	8.1 × 10^−6^	2410	4.24	1750	4.6×10^-9^	[34]
Monomeric NAD-IDH								
*Campylobacter* sp. NAD-IDH (Mg^2+^)	28.9	7.0	0.242	513.2	1.9	0.004	61	[12]
*C. curvus* NAD-IDH (Mg^2+^) ^a^	74.2	10.8	0.146	475.9	2.0	0.004	37	[12]

^a^ *S. suis*, *Streptococcus suis*; *C. curvus*, *Campylobacter curvus*; “-” not detectable; ^b^ Engineered *E. coli* NAD-IDH: K344D/Y345I/V351A/Y391K/R395S/C332Y/C201M; Engineered *B. longum* NAD-IDH: D253A/S257K/K260Q/R314D/H315I/T327A; Engineered *Y. lipolytica* NAD-IDH: R322D; ^c^ A, *k*_cat_/*K*_m_^NAD^; B, *k*_cat_/*K*_m_^NADP^.

## Supplementary Materials

Supplementary materials can be found at http://www.mdpi.com/1422-0067/17/3/296/s1.

## Abbreviations

PCR: polymerase chain reaction
LB: Luria-Bertani
IPTG: isopropyl-1-thio-β-d-galactopyranoside
SDS-PAGE: sodium dodecyl sulfate polyacrylamide gel electrophoresis
CD: circular dichroism
SEC: size exclusion chromatography
IDH: isocitrate dehydrogenase
NADP^+^: nicotinamide adenine dinucleotide phosphate
NAD^+^: nicotinamide adenine dinucleotide
NADP-IDH: NADP^+^-dependent isocitrate dehydrogenase
NAD-IDH: NAD^+^-dependent isocitrate dehydrogenase
*Bl*IDH: isocitrate dehydrogenase from *Bifidobacterium longum*
*Ec*IDH: NADP-IDH from *Escherichia coli*
*Hc*IDH: human cytosolic NADP-IDH
*At*IDH: NAD-IDH from *Acidithiobacillus thiooxidans*
*Ot*IDH: NAD-IDH from *Ostreococcus tauri*
*K*_m_: Michaelis constant
*k*_cat_: catalytic rate constant

## References

[1] Sivaraman J., Li Y., Banks J., Cane D.E., Matte A., Cygler M. (2003). Crystal structure of *Escherichia coli* PdxA, an enzyme involved in the pyridoxal phosphate biosynthesis pathway. J. Biol. Chem..

[2] Miyazaki J., Kobashi N., Nishiyama M., Yamane H. (2003). Characterization of homoisocitrate dehydrogenase involved in lysine biosynthesis of an extremely thermophilic bacterium, *Thermus thermophilus* HB27, and evolutionary implication of β-decarboxylating dehydrogenase. J. Biol. Chem..

[3] Tipton P.A., Beecher B.S. (1994). Tartrate dehydrogenase, a new member of the family of metal-dependent decarboxylating R-hydroxyacid dehydrogenases. Arch. Biochem. Biophys..

[4] Dean A.M., Koshland D.E. (1993). Kinetic mechanism of *Escherichia coli* isocitrate dehydrogenase. Biochemistry.

[5] Fernie A.R., Carrari F., Sweetlove L.J. (2004). Respiratory metabolism: Glycolysis, the TCA cycle and mitochondrial electron transport. Curr. Opin. Plant Biol..

[6] Spaans S.K., Weusthuis R.A., van der Oost J., Kengen S.W. (2015). NADPH-generating systems in bacteria and archaea. Front. Microbiol..

[7] Lee S.M., Koh H.J., Park D.C., Song B.J., Huh T.L., Park J.W. (2002). Cytosolic NADP^+^-dependent isocitrate dehydrogenase status modulates oxidative damage to cells. Free Radic. Biol. Med..

[8] Jo S.H., Son M.K., Koh H.J., Lee S.M., Song I.H., Kim Y.O., Lee Y.S., Jeong K.S., Kim W.B., Park J.W. (2001). Control of mitochondrial redox balance and cellular defense against oxidative damage by mitochondrial NADP^+^-dependent isocitrate dehydrogenase. J. Biol. Chem..

[9] Cohen A.L., Holmen S.L., Colman H. (2013). IDH1 and IDH2 mutations in gliomas. Curr. Neurol. Neurosci. Rep..

[10] Chen C., Liu Y., Lu C., Cross J.R., Morris J.P., Shroff A.S., Ward P.S., Bradner J.E., Thompson C., Lowe S.W. (2013). Cancer-associated IDH2 mutants drive an acute myeloid leukemia that is susceptible to Brd4 inhibition. Genes Dev..

[11] Dang L., White D.W., Gross S., Bennett B.D., Bittinger M.A., Driggers E.M., Fantin V.R., Jang H.G., Jin S., Keenan M.C. (2009). Cancer-associated IDH1 mutations produce 2-hydroxyglutarate. Nature.

[12] Wang P., Lv C., Zhu G. (2015). Novel type II and monomeric NAD^+^ specific isocitrate dehydrogenases: Phylogenetic affinity, enzymatic characterization, and evolutionary implication. Sci. Rep..

[13] Tang W.G., Song P., Cao Z.Y., Wang P., Zhu G.P. (2015). A unique homodimeric NAD^+^-linked isocitrate dehydrogenase from the smallest autotrophic eukaryote *Ostreococcus tauri*. FASEB J..

[14] Peng Y., Zhong C., Huang W., Ding J. (2008). Structural studies of *Saccharomyces cerevesiae* mitochondrial NADP-dependent isocitrate dehydrogenase in different enzymatic states reveal substantial conformational changes during the catalytic reaction. Protein Sci..

[15] Xu X., Zhao J., Xu Z., Peng B., Huang Q., Arnold E., Ding J. (2004). Structures of human cytosolic NADP-dependent isocitrate dehydrogenase reveal a novel self-regulatory mechanism of activity. J. Biol. Chem..

[16] Ceccarelli C., Grodsky N.B., Ariyaratne N., Colman R.F., Bahnson B.J. (2002). Crystal structure of porcine mitochondrial NADP^+^-dependent isocitrate dehydrogenase complexed with Mn^2+^ and isocitrate. Insights into the enzyme mechanism. J. Biol. Chem..

[17] Zhu G., Golding G.B., Dean A.M. (2005). The selective cause of an ancient adaptation. Science.

[18] Srutkova D., Spanova A., Spano M., Drab V., Schwarzer M., Kozakova H., Rittich B. (2011). Efficiency of PCR-based methods in discriminating *Bifidobacterium longum* ssp. longum and *Bifidobacterium longum* ssp. *infantis* strains of human origin. J. Microbiol. Methods.

[19] Yuan J., Zhu L., Liu X., Li T., Zhang Y., Ying T., Wang B., Wang J., Dong H., Feng E. (2006). A proteome reference map and proteomic analysis of *Bifidobacterium longum* NCC2705. Mol. Cell. Proteom..

[20] Schell M.A., Karmirantzou M., Snel B., Vilanova D., Berger B., Pessi G., Zwahlen M.C., Desiere F., Bork P., Delley M. (2002). The genome sequence of *Bifidobacterium longum* reflects its adaptation to the human gastrointestinal tract. Proc. Natl. Acad. Sci. USA.

[21] Lin M.Y., Chang F.J. (2000). Antioxidative effect of intestinal bacteria *Bifidobacterium longum* ATCC 15708 and *Lactobacillus acidophilus* ATCC 4356. Dig. Dis. Sci..

[22] Iwabuchi N., Xiao J.Z., Yaeshima T., Iwatsuki K. (2011). Oral administration of *Bifidobacterium longum* ameliorates influenza virus infection in mice. Biol. Pharm. Bull..

[23] Xiao J.Z., Kondo S., Yanagisawa N., Miyaji K., Enomoto K., Sakoda T., Iwatsuki K., Enomoto T. (2007). Clinical efficacy of probiotic *Bifidobacterium longum* for the treatment of symptoms of Japanese cedar pollen allergy in subjects evaluated in an environmental exposure unit. Allergol. Int..

[24] Sivan A., Corrales L., Hubert N., Williams J.B., Aquino-Michaels K., Earley Z.M., Benyamin F.W., Lei Y.M., Jabri B., Alegre M.L. (2015). Commensal *Bifidobacterium* promotes antitumor immunity and facilitates anti-PD-L1 efficacy. Science.

[25] Yazawa K., Fujimori M., Amano J., Kano Y., Taniguchi S. (2000). *Bifidobacterium longum* as a delivery system for cancer gene therapy: Selective localization and growth in hypoxic tumors. Cancer Gene Ther..

[26] Imada K., Tamura T., Takenaka R., Kobayashi I., Namba K., Inagaki K. (2008). Structure and quantum chemical analysis of NAD^+^-dependent isocitrate dehydrogenase: Hydride transfer and co-factor specificity. Proteins.

[27] Zhao X., Wang P., Zhu G., Wang B., Zhu G. (2014). Enzymatic characterization of a type II isocitrate dehydrogenase from pathogenic *Leptospira interrogans* serovar Lai strain 56601. Appl. Biochem. Biotechnol..

[28] Chen R., Yang H. (2000). A highly specific monomeric isocitrate dehydrogenase from *Corynebacterium glutamicum*. Arch. Biochem. Biophys..

[29] Kanao T., Kawamura M., Fukui T., Atomi H., Imanaka T. (2002). Characterization of isocitrate dehydrogenase from the green sulfur bacterium *Chlorobium limicola*. Eur. J. Biochem..

[30] Wu M.C., Tian C.Q., Cheng H.M., Xu L., Wang P., Zhu G.P. (2015). A novel type II NAD^+^-specific isocitrate dehydrogenase from the marine bacterium *Congregibacter litoralis* KT71. PLoS ONE.

[31] Wang P., Jin M., Zhu G. (2012). Biochemical and molecular characterization of NAD^+^-dependent isocitrate dehydrogenase from the ethanologenic bacterium *Zymomonas mobilis*. FEMS Microbiol. Lett..

[32] Dean A.M., Golding G.B. (1997). Protein engineering reveals ancient adaptive replacements in isocitrate dehydrogenase. Proc. Natl. Acad. Sci. USA.

[33] Jennings G.T., Minard K.I., McAlister-Henn L. (1997). Expression and mutagenesis of mammalian cytosolic NADP^+^-specific isocitrate dehydrogenase. Biochemistry.

[34] Li X., Wang P., Ge Y., Wang W., Abbas A., Zhu G. (2013). NADP^+^-specific isocitrate dehydrogenase from oleaginous yeast *Yarrowia lipolytica* CLIB122: Biochemical characterization and coenzyme sites evaluation. Appl. Biochem. Biotechnol..

[35] Huang D., Liu J., Shen G. (2009). Cloning, expression, and enzymatic characterization of isocitrate dehydrogenase from *Helicobacter pylori*. Protein J..

[36] Watanabe S., Yasutake Y., Tanaka I., Takada Y. (2005). Elucidation of stability determinants of cold-adapted monomeric isocitrate dehydrogenase from a psychrophilic bacterium, *Colwellia maris*, by construction of chimeric enzymes. Microbiology.

[37] Hurley J.H., Chen R., Dean A.M. (1996). Determinants of cofactor specificity in isocitrate dehydrogenase: Structure of an engineered NADP^+^→NAD^+^ specificity-reversal mutant. Biochemistry.

[38] Rodriguez-Arnedo A., Camacho M., Llorca F., Bonete M.J. (2005). Complete reversal of coenzyme specificity of isocitrate dehydrogenase from *Haloferax volcanii*. Protein J..

[39] Wang P., Jin M., Su R., Song P., Wang M., Zhu G. (2011). Enzymatic characterization of isocitrate dehydrogenase from an emerging zoonotic pathogen *Streptococcus suis*. Biochimie.

[40] Steen I.H., Lien T., Madsen M.S., Birkeland N.K. (2002). Identification of cofactor discrimination sites in NAD-isocitrate dehydrogenase from *Pyrococcus furiosus*. Arch. Microbiol..

[41] Tamura K., Stecher G., Peterson D., Filipski A., Kumar S. (2013). MEGA6: Molecular evolutionary genetics analysis version 6.0. Mol. Biol. Evol..

[42] Bramucci E., Paiardini A., Bossa F., Pascarella S. (2012). PyMod: Sequence similarity searches, multiple sequence-structure alignments, and homology modeling within PyMOL. BMC Bioinform..

[43] Pettersen E.F., Goddard T.D., Huang C.C., Couch G.S., Greenblatt D.M., Meng E.C., Ferrin T.E. (2004). UCSF Chimera—A visualization system for exploratory research and analysis. J. Comput. Chem..

[44] Ho S.N., Hunt H.D., Horton R.M., Pullen J.K., Pease L.R. (1989). Site-directed mutagenesis by overlap extension using the polymerase chain-reaction. Gene.

[45] Pace C.N., Vajdos F., Fee L., Grimsley G., Gray T. (1995). How to measure and predict the molar absorption coefficient of a protein. Protein Sci..

[46] Cvitkovitch D.G., Gutierrez J.A., Bleiweis A.S. (1997). Role of the citrate pathway in glutamate biosynthesis by *Streptococcus mutans*. J. Bacteriol..

