# Supplementary material for: Biochemical Characterization and Complete Conversion of Coenzyme Specificity of Isocitrate Dehydrogenase from Bifidobacterium longum
Source: Int J Mol Sci. 2016 Feb 26;17(3):296. doi: 10.3390/ijms17030296 (PMC4813160; doi:10.3390/ijms17030296)
Supplement: Supplementary file 1 [file ijms-17-00296-s001.pdf]

Supplementary Materials: Biochemical Characterization and Complete Conversion of Coenzyme Specificity of Isocitrate Dehydrogenase from *Bifidobacterium longum*

Shi-Ping Huang, Hong-Mei Cheng, Peng Wang and Guo-Ping Zhu

Table S1. Comparison of kinetic parameters between the recombinant *Bl*IDH and other IDHs. "-" not determined.

Species	NADP⁺			NAD⁺			Type	Reference
	K_m (μM)	k_{cat} (s^{-1})	k_{cat}/K_m ($s^{-1}·μM^{-1}$)	K_m (μM)	k_{cat} (s^{-1})	k_{cat}/K_m ($s^{-1}·μM^{-1}$)		
*Bl*IDH (Mn^{2+})	58.29	48.19	0.83	552.2	2.37	0.0043	Type II	This study
*Bl*IDH (Mg^{2+})	19.45	36.38	1.87	3584	11.70	0.0033	Type II	This study
L. interrogans IDH (Mg^{2+})	21.1	22.32	1.21	8983	1.739	0.000193	Type II	[27]
Y. lipolytica IDH (Mg^{2+})	59 ± 9.1	72 ± 8.8	1.22	-	-	-	Type II	[34]
R. norvegicus IDH (cytosolic) (Mg^{2+})	11.5	88.2	9.1	-	-	-	Type II	[33]
E. coli IDH (Mg^{2+})	17	80.5	4.7	4700	3.22	0.00069	Type I	[32]
H. pylori IDH (Mg^{2+})	176	124	0.704	-	-	-	Type I	[35]
C. glutamicum IDH (Mn^{2+})	4	87	21.75	1900 0	9.3	0.00049	Monomeric	[28]
C. maris IDH (Mg^{2+})	6.9	61.3	8.9	-	-	-	Monomeric	[36]

Table S2. Primers for amplification of wild-type and mutant *Bl*IDH genes.

Primers	Nucleotide Sequences 5′ to 3′
*Bl*IDH-S [a]	GCGAATCATATGGCCAAAATCAAGGTCGAAGG
*Bl*IDH-As [a]	TACCGCTCGAGTTACTCGGCCAGAGCCTT
*Bl*IDH-R314D-S	CACCGTGACC<u>GAC</u>CACTACCGCCG
*Bl*IDH-R314D-As	CGGCGGTAGTG<u>GTC</u>GGTCACGGTG
*Bl*IDH-R314D/H315I-S	GCACCGTGACC<u>GACATC</u>TACCGCCGCTG
*Bl*IDH-R314D/H315I-As	GCGGCGGTA<u>GATGTC</u>GGTCACGGTG
*Bl*IDH-T327A-S	GGCAGAAGGGCGAGAAGACCTCC<u>GCC</u>AACCCGATCGCCTCCATCTTC
*Bl*IDH-T327A-As	GAAGATGGAGGCGATCGGGTT<u>GGC</u>GGAGGTCTTCTCGCCCTTC
*Bl*IDH-R314D/H315M-S	CGCACGGCACCGTGACC<u>GATATG</u>TACCGCCGCTGGCAGAAG
*Bl*IDH-R314D/H315M-As	CTTCTGCCAGCGGCGGTA<u>CATATC</u>GGTCACGGTGCCGTGCG
*Bl*IDH-T327L-S	GCGAGAAGACCTCC<u>CTG</u>AACCCGATCGCCTCC
*Bl*IDH-T327L-As	GGAGGCGATCGGGTT<u>CAG</u>GGAGGTCTTCTCGC
*Bl*IDH-D253A/S257K/K260Q-S	CACCGCCTGATCGAC<u>GCC</u>ATGGTGGCC<u>AAG</u>TCCCTG<u>CAA</u>TGGCACGGCGGTTAC
*Bl*IDH-D253A/S257K/K260Q-As	GTAACCGCCGTGCCA<u>TTG</u>CAGGGA<u>CTT</u>GGCCACCAT<u>GGC</u>GTCGATCAGGCGGTG

[a] "S" and "As": indicate the sense (S) and antisense (As) primers of the corresponding genes. Underlined bases indicate the mutant sites.

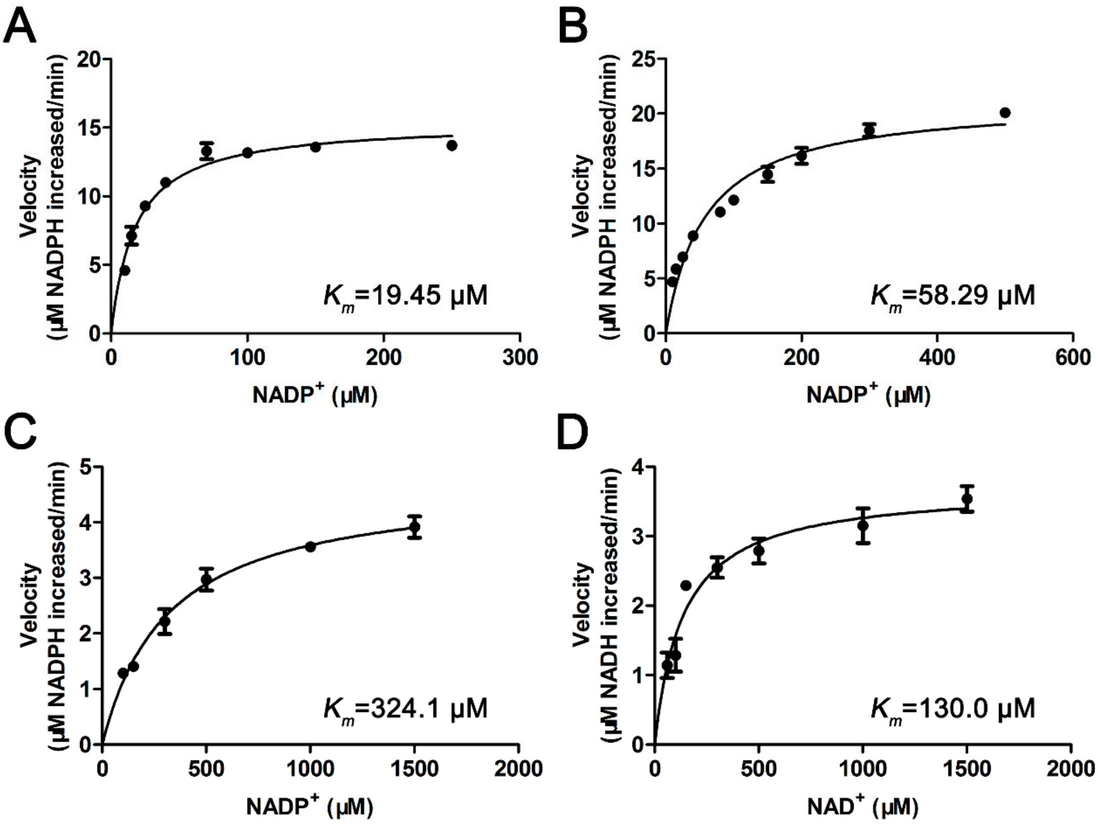

Figure S1. Kinetic analyses of the wile-type and sextuple mutant *Bl*IDH. The *Bl*IDH K_m for $NADP^+$ were 19.45 and 58.29 μM with Mg^{2+} (**A**) and Mn^{2+} (**B**), respectively; The sextuple mutant *Bl*IDH K_m for $NADP^+$ (**C**) and NAD^+ (**D**) were 324.1 and 130.0 μM, respectively.

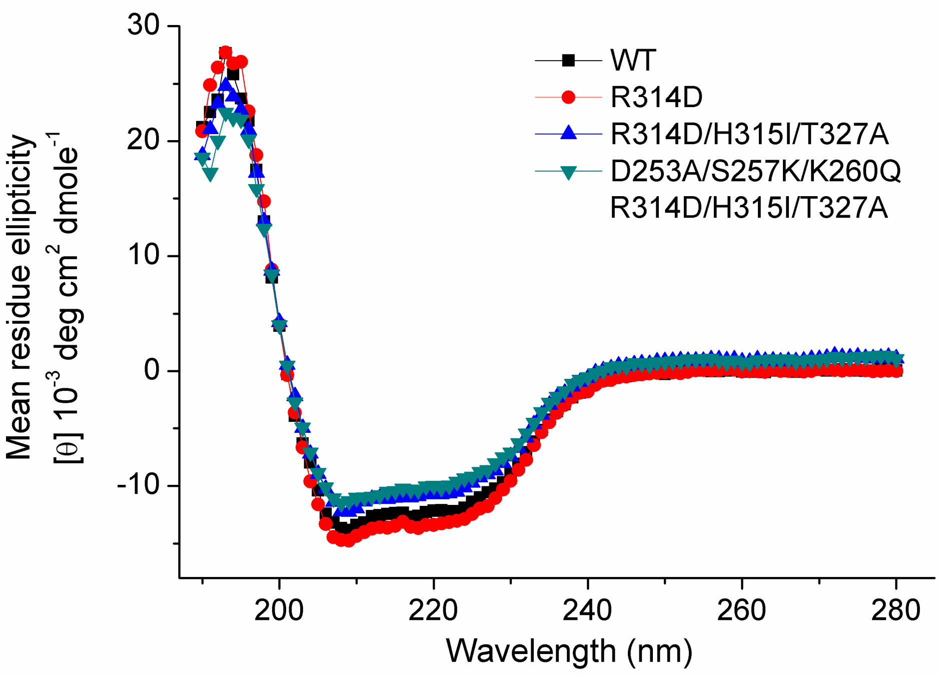

Figure S2. Circular dichroism (CD) spectra of the wild-type *Bl*IDH and its mutants.
